# Diversity of *nifH* Gene in Culturable Rhizobia from Black Locust (*Robinia pseudoacacia* L.) Grown in Cadmium-Contaminated Soils

**DOI:** 10.3390/biology14040362

**Published:** 2025-03-31

**Authors:** Xiaomeng Wang, Xia Jia, Yonghua Zhao, Yuan Xie, Xiuxin Meng, Fang Wang

**Affiliations:** 1 Key Laboratory of Subsurface Hydrology and Ecological Effects in Arid Region of Ministry of Education, Shaanxi Key Laboratory of Land Consolidation, Key Laboratory of Eco-Hydrology and Water Security in Arid and Semi-Arid Regions of Ministry of Water Resources, School of Water and Environment, Chang’an University, Xi’an 710054, China; 2022129071@chd.edu.cn (X.W.);; 2 School of Land Engineering, Chang’an University, Xi’an 710054, China

**Keywords:** cadmium pollution, black locust, culturable rhizobia, *nifH* gene, diversity

## Abstract

(1) Background: Rhizobia can promote plant growth by providing essential nutrients such as NH_4_^+^ and PO_4_^3−^; thus, rhizobia that can tolerate the stress of heavy metals will be conducive to the phytoremediation of heavy-metal-contaminated soils. Therefore, understanding the dominant heavy-metal-tolerant rhizobia that can be cultured is important for the establishment of an indigenous legume–rhizobia symbiotic remediation system; (2) Methods: Here, we investigated *nifH* gene diversity in culturable rhizobia from black locust (*Robinia pseudoacacia* L.) grown in cadmium (Cd)-contaminated soils using high-throughput sequencing.; (3) Results: A total of 16 genera and 26 species were identified from the cultures of root nodules of black locust exposed to five Cd levels. Cadmium did not show a significant effect on the abundance, diversity, and evenness of the culturable rhizobia community. However, Cd significantly affected the community structure of culturable rhizobia containing *nifH. Mesorhizobium, Sinorhizobium*, and *Rhizobium* were the absolute dominant genera present in the cultures under five Cd treatments. Additionally, Cd significantly affected the relative abundance of *Azohydromonas, Xanthobacter, Skermanella, Bradyrhizobium, Paenibacillus,* and *Pseudacidovorax* in the cultures. Soil pH, total Cd, DTPA-Cd, and C/H ratio were the significant factors on culturable rhizobia community.; (4) Conclusions: Cd showed a negative effect on *nifH* gene community of culturable rhizobia from black locust, which will provide insight into the selection of excellent strains that can promote phytoremediation of heavy-metal-contaminated soils.

## Simple Summary

This study investigated culturable rhizobia diversity in black locust (*Robinia pseudoacacia* L.) grown in cadmium (Cd)-contaminated soils to identify metal-tolerant strains for enhancing phytoremediation. Using high-throughput sequencing of the *nifH* gene, 16 genera and 26 species were identified across five Cd treatments. Cd exposure did not significantly alter rhizobia abundance, diversity, or evenness but reshaped the *nifH*-containing community structure. Dominant genera (*Mesorhizobium*, *Sinorhizobium*, *Rhizobium*) persisted under all Cd levels, while the relative abundance of *Azohydromonas*, *Xanthobacter*, and others shifted significantly. Soil pH, total Cd, DTPA-Cd, and C/H ratio were key factors influencing community composition. Although Cd negatively affected the *nifH* gene community, the resilience of dominant rhizobia highlights their potential for symbiotic remediation systems. These findings aid in selecting stress-tolerant rhizobia to optimize plant growth and metal detoxification in contaminated soils.

## 1. Introduction

Rhizobia from legumes are capable of converting atmospheric nitrogen to the nitrogen that plants can absorb, such as ammonia and nitrate nitrogen [1,2], which is an environmentally friendly way to supply nitrogen to the terrestrial ecosystem [3]. Therefore, the use of rhizobia has great potentiality in reducing the use of artificial nitrogen fertilizer, increasing yields and sustainable agricultural development [4,5]. Especially, rhizobia that can tolerate the stress of heavy metals will be conducive to the phytoremediation of heavy-metal-contaminated soils by promoting plant growth [6,7]. Relative to other physicochemical methods, the symbiosis system that rhizobia forms with the roots of legume plants is economical, effective, and environmentally friendly in the restoration of heavy-metal-contaminated soils [8,9], which is receiving increasing attention. Exotic strains that can remediate polluted soils possibly cause a negative competition between them and indigenous microbial communities [10], which sometimes might lead to some ecological risks to local environments. However, the application of an indigenous legume plants–rhizobium symbiosis system can avoid possible ecological risks caused by exotic strains in remediating polluted environments. Most studies analyzed rhizobial diversity by directly extracting nodule DNA [11]; however, understanding the diversity of culturable rhizobia might be more meaningful for the remediation of polluted soils than root nodule DNA analysis. Zhao [12] et al. and Zheng [11] et al. found that *Sinorhizobium* and *Rhizobium* were present in both cultured and non-cultured rhizobial from wild soybean; however, *Enterobacter*, *Stenotrophomonas*, and *Chryseobacterium* in non-cultured rhizobia were not found in the cultures, which suggested that culturable rhizobia might be vital for the practical application. Thus, the understanding of dominant heavy-metal-tolerant rhizobia that can be cultured is important for the establishment of an indigenous legume plant–rhizobia symbiotic remediation system. However, the study on this topic was still scarce at present.

Due to its high toxicity, long persistence, bioconcentration, and high mobility [13,14,15], cadmium (Cd) is a non-essential and toxic element for all living organisms [16,17,18]. Additionally, lots of studies have indicated that Cd can affect soil fertility by reducing abundance, diversity, and activity of soil microorganisms, inhibiting soil respiration, and decreasing bio-mineralization ability of soils [19,20,21], which suggests that Cd-contaminated soils needs to be urgently remediated.

**Figure 1:**
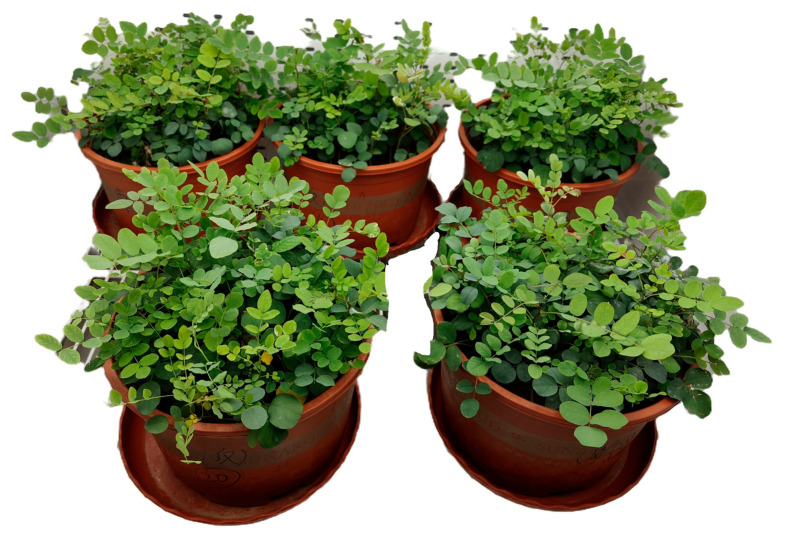
Picture of black locusts.

As nitrogen-fixing woody legume by forming symbiotic systems with rhizobia like *Mesorhizobium amorphae* [22], black locust (*Robinia pseudoacacia* L.) (Figure 1) has fast-growing capability, resistance to nutrient depletion and heavy metals, deeper root system, and economic value, and it is widely planted to restore degraded ecosystems [22,23,24,25]. Furthermore, Hao [22] found that *Mesorhizobium amorphae* can enhance the uptake of copper by black locusts grown in copper-contaminated soils. Overall, it is important for phytoremediation of heavy-metal-contaminated soils to understand the diversity of rhizobia isolated from plant roots. Considering the effect of heavy metals in soils on a rhizobia-plant symbiotic fixation system [26], the study aimed to investigate the effect of Cd in soils on the community structure of culturable indigenous nitrogen-fixing rhizobia that could colonize plant roots. The results will provide a meaningful insight into the selection of rhizobia that can fix nitrogen and resist heavy metal stress.

## 2. Materials and Methods

### 2.1. Soil Preparation and Plant Seeds

The soils for the experiment were collected in Huoditang, Qinling Mountains, China (33°18′–34°26′ N, 106°04′–110°40′ E), and the soil type is brown forest soil (WRB-based soil classification, 1998). Soil pH was 6.28. Soil total C, N, and S content was 4.69 mg/kg, 3.90 mg/kg, and 0.01 mg/kg, respectively; C/N ratio was 12.01; and total Cd was 0.81 mg/kg. To obtain culturable rhizobia community or strains that can survival in Cd-contaminated soils, we used black locust grown in Cd-contaminated soils to screen N-fixing nodules according to previous studies [27]. The prepared five Cd levels were Cd1 (2 mg Cd kg^−1^ dry weight soil), Cd2 (4 mg Cd kg^−1^ dry weight soil), Cd3 (7 mg Cd kg^−1^ dry weight soil), Cd4 (15 mg Cd kg^−1^ dry weight soil), and Cd5 (20 mg Cd kg^−1^ dry weight soil), respectively. The soils treated with 3CdSO_4_·8H_2_O solution were placed in the dark to balance for one month and were stirred by a shovel once a week.

Black locust seeds were obtained from the Academy of Forestry, Northwest A&F University, Yangling, China.

### 2.2. Pot Experiments and Soil and Nodule Sampling

First, 3.5 kg of the above-treated soils was placed in each pot (15 cm diameter × 20 cm height). Black locust seeds were sown at ~1.5 cm layer of soils, and then, all pots were placed in plant incubators (Percival E-36 L, Percival Scientific, Perry, IA, USA) with an accurate temperature and humidity regulator. The average temperature in the incubator was set to 21.5 °C (according to 25 °C and 18 °C during day and night, respectively), and humidity was maintained at 65–69% during the experiment. The average light intensity was 550 μmol m^−2^s^−1^ over a 12 h light cycle. Twenty seedlings per pot were selected for the experiment after emergence, and weeds in the pots were removed by hand during the experiment. Three replicates were prepared for each treatment.

The black locust cultured soils were gently shaken off from the roots for the determination of soil physiochemical properties after 90-day growth, and the nodules with partial roots were cut off using scissors for the analysis of rhizobia.

### 2.3. Determination of Soil Physiochemical Characteristics

Soil pH was determined by a pH meter (Mettler Toledo S220, Mettler Toledo, Zurich, Switzerland) according to a 2.5:1 ratio of water to soil. The C, N, and S contents and C/N ratio were determined by an elemental analyzer (Vario Macrocube, Hanau, Elementar, Germany) using active soil constituents (GBW07460, ASA-9, Shaanxi Huangmian Soil Composition Analysis Standard Reference Material, China) as a standard substance. Total Cd and DTPA-Cd contents were determined by graphite furnace atomic absorption spectrophotometry as described by Li [28]. Moreover, 0.2 g of soils were digested in a mixture of H_2_O_2_ and HNO_3_ (8: 1, *v*:*v*) by a microwave digester (WX-6000, Shanghai Yiyao, Shanghai, China). The total Cd in the digestive solution was analyzed by atomic absorption spectrophotometry (AAS, AA-7020, East-West Analysis, Beijing, China) as described by Li [28]. The available Cd in soils was determined by AAS Exact after 10 g of soils was extracted with diethylenetriamine pentaacetic acid (DTPA).

### 2.4. Rhizobial Isolation

The large and full nodules were soaked in 95% ethanol for 5 min to remove surface tension after being soaked in distilled water for 5 min to remove impurities. Then, the nodules were soaked in 0.1% HgCl_2_ solution for 5 min after the ethanol was removed completely. Finally, the nodules were rinsed with sterile water 15 times. The nodules were punctured with sterile pointed forceps in 200 µL of 0.9% (m/v) NaCl, and then the traumatized surface was attached to YMA medium (containing 5 mL of 0.5% (v/m) Congo red, 10 g of mannitol, 3 g of yeast powder, 0.1 g of NaCl, 0.2 g of MgSO_4_·7H_2_O, 0.25 g of K_2_HPO_4_, 0.25 g of KH_2_PO_4_, 15 g of agar, and 1000 mL of distilled water with a pH = 6.8–7.0). All the round and creamy-white colonies were obtained for the analysis of diversity of the *nifH* gene after the nodules were incubated for 4 days at 28 °C according to previous studies [29,30].

### 2.5. Analysis of nifH Gene Diversity in Culturable Rhizobia

The *nifH* gene diversity of rhizobium was determined through the Illumina Miseq platform (Illumina Inc., San Diego, CA, USA). A bacterial genomic DNA extraction kit (Solarbio, Beijing, China) was used to extract DNA of rhizobium colonies. Target DNA fragments of *nifH* gene were amplified by PCR using the primers (PolF: 5′-TGCGAYCCSAARGCBGACTC3′, PolR: 5′-ATSGCCATCATYTCRCCGGA-3′). The PCR products were sequenced after being checked for quality by 2% agarose gel electrophoresis and quantified using the Quant-iT PicoGreen dsDNA Detection Kit after purification through a DNA extraction kit (Axygen, Union City, CA, USA). After the primer fragments in the original sequences were cut using cutadapt (v2.3), the sequences were spliced and quality controlled by removing duplicates and chimeras using the Vsearch software (v2.13.4_linux_x86_64). Thus, the high-quality sequences were easily obtained. QIIME software (version 1.8.0, http://qiime.org/ (accessed on 21 September 2024)) was used to output the operational taxonomic units (OTUs) by clustering the high-quality sequences at 97% similarity level, and R 4.1.1 software was used to plot the Venn diagrams. The representative sequences with higher relative abundance were annotated using the NT database (2019.8, http://ftp.ncbi.nih.gov/blast/db/ (accessed on 21 September 2024)) to obtain the corresponding taxonomic information. Principal coordinate analysis (PCoA), non-metric multidimensional scaling analysis (NMDS), and cluster analysis were used to estimate the beta diversity of *nifH* in the culturable rhizobia community by the unweighted pair-group method with arithmetic means (UPGMA). Linear discriminant analysis (Lefse) effect sizes were applied to evaluate genus-level relative abundance matrices via the Galaxy online platform (http://huttenhower.sph.harvard.edu/galaxy/ (accessed on 21 September 2024)), and then linear discriminant analysis (LDA) effect size (Lefse) analysis according to the relative abundance (LDA > 2) was carried out to reduce the dimensionality of the data in assessing the effects of species with significant divergence.

### 2.6. Statistical Analysis

QIIME 2 software was used to evaluate α-diversity (Chao1, Goods_coverage, Observed_species, Shannon and Simpson, Pielou_evenness_index) of the *nifH* gene in culturable rhizobia. The relationship between the *nifH* gene in culturable rhizobia from nodules and soil physicochemical properties was analyzed by redundancy analysis (RDA) using Canoco software (5.0). Pearson correlation coefficients were used to analyze the relationship between culturable dominant rhizobia and soil physicochemical properties by using SPSS software 24.0. The effect of Cd on soil parameters and diversity indices of *nifH* was analyzed by one-way analysis of variance using SPSS software 24.0.

## 3. Results

### 3.1. Soil Physiochemical Characteristics

**Figure 2:**
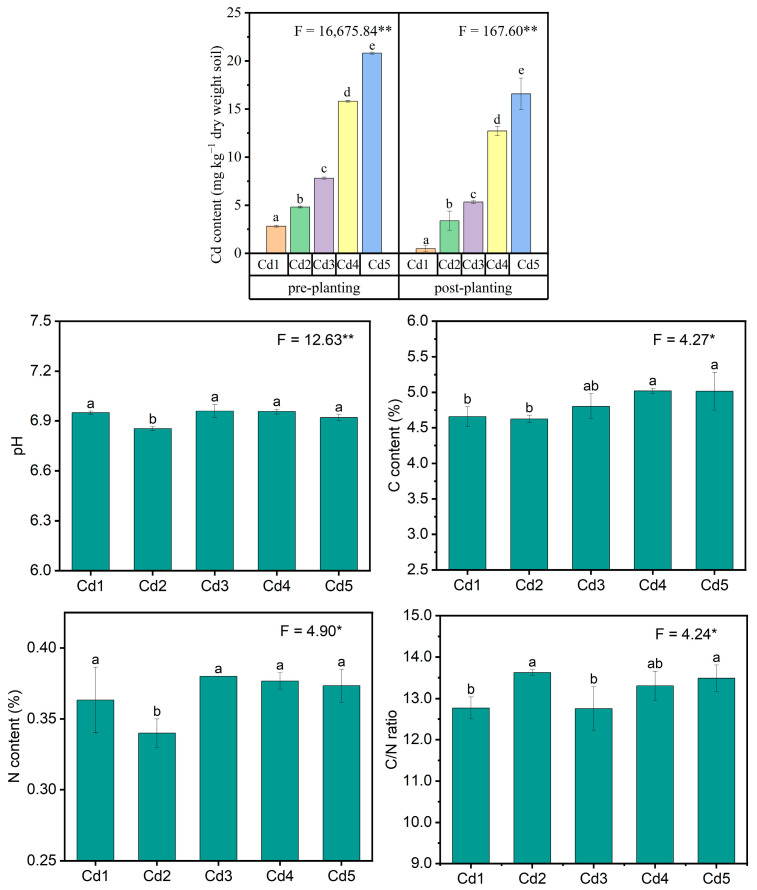

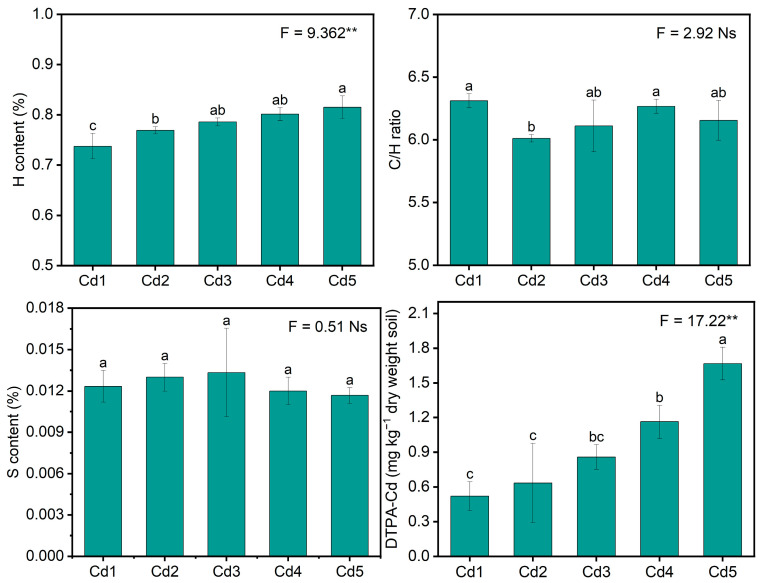
Total Cd, pH, TC, TN, C/N ratio, H, C/H ratio, TS, and DTPA-Cd in soils cultured with black locust under Cd exposure tested by one-way factorial analysis (ANOVA) and summary of the ANOVA results (F values and significance levels). Results were means ± SE (*n* = 3). Cd1–Cd5 represent 2, 4, 7, 15, and 20 mg Cd kg^−1^ dry weight soil, respectively. Different lowercase letters indicate significant divergence (*p* < 0.05) between different treatments. The * and ** represented significance at *p* < 0.05 and *p* < 0.01, respectively, and Ns represents not significant.

The total Cd in soils under all treatments significantly decreased relative to the designed concentration (Figure 2). The soil pH slightly increased relative to the original soil pH (6.28), and the divergence between different treatments was insignificant except for Cd2 treatment with the smallest (*p* < 0.05) pH (Figure 2). Soil TC increased (*p* < 0.05) under Cd4 and Cd5 treatments compared with treatments of Cd1 and Cd2 (Figure 2). Soil TN was the lowest (*p* < 0.05) at Cd2 level (Figure 2). The C/N ratio of soils increased (*p* < 0.05) under Cd2 treatment relative to treatments of Cd1 and Cd3 (Figure 2). The H content in soils significantly increased under Cd2-Cd5 levels relative to Cd1 and was the highest under Cd5 treatment (Figure 2). The C/H ratio decreased (*p* < 0.05) at Cd2 level compared to treatments of Cd1 and Cd4 (Figure 2). However, the changes in soil TS were insignificant (Figure 2). The difference in DTPA-Cd was insignificant between Cd1, Cd2, and Cd3 and the highest at Cd5 level, which showed an increasing trend with Cd levels (Figure 2).

### 3.2. Sample Sequencing Quality

**Figure 3:**
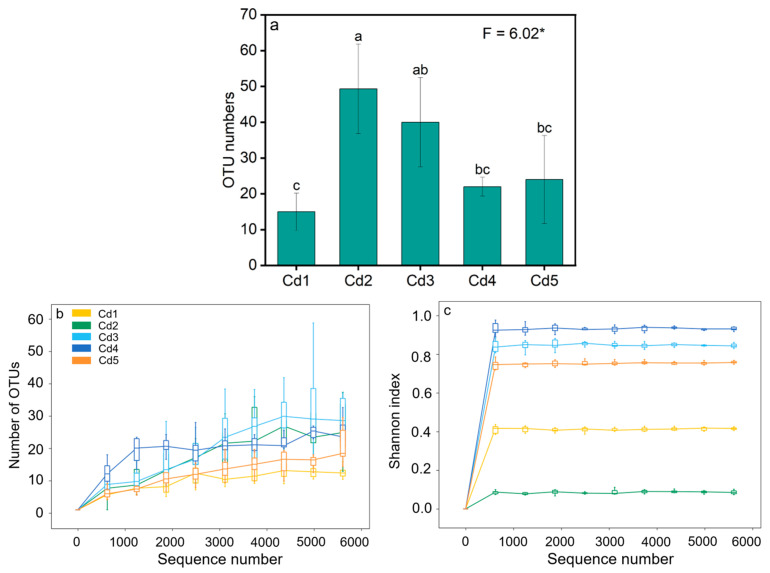
The operational taxonomic unit (OTU) numbers (**a**), OTU rarefaction curves (**b**), and Shannon index rarefaction curves (**c**) of culturable rhizobia from black locust grown in Cd-contaminated soils. Different lowercase letters indicate significant difference (*p* < 0.05) between different treatments. Cd1–Cd5 represent 2, 4, 7, 15, and 20 mg Cd kg^−1^ dry weight soil, respectively. The * represents significance at *p* < 0.05.

A total of 1,275,712 original sequences from all treatments were obtained, and 1,163,757 high-quality sequences were selected after splicing and effective processing. Finally, 77,584 high-quality sequences were obtained averagely from the culturable rhizobia. A total of 119 OTUs were identified after the high-quality sequences were clustered and denoised at a 97% similarity. The OTUs of culturable rhizobia were 15, 49, 40, 22, and 24 under Cd1–Cd5, respectively, and the difference in OTU number between Cd1 and Cd2 was significant (Figure 3a). Additionally, the *nifH* gene in culturable rhizobia from black locust grown in Cd-contaminated soils was classified into 2 phylum, 4 classes, 7 orders, 12 families, 16 genera, and 26 species according to the similarity of OTUs at 97% level. The rarefaction and Shannon index curves were used to verify *nifH* gene richness when the sequence number under all treatments was different, and the *nifH* genes detected by sequencing increased gradually with sequencing volume. When the sequencing data reached 5000, the data on the samples tended to flatten out, and the OTU data no longer increased dramatically. Thus, the number of sequences under all treatments was reasonable, and no further new OTUs would be added along with increasing sequencing data (Figure 3b). The Shannon index curve under all treatments was saturated, which indicated that the cultures were reasonable (Figure 3c).

### 3.3. Alpha Diversity of nifH Genes in Culturable Rhizobia Community

**Figure 4:**
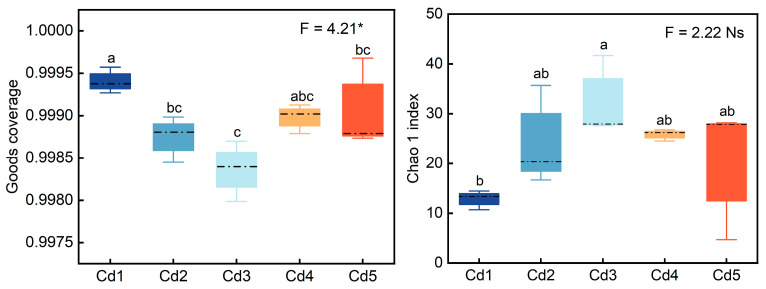

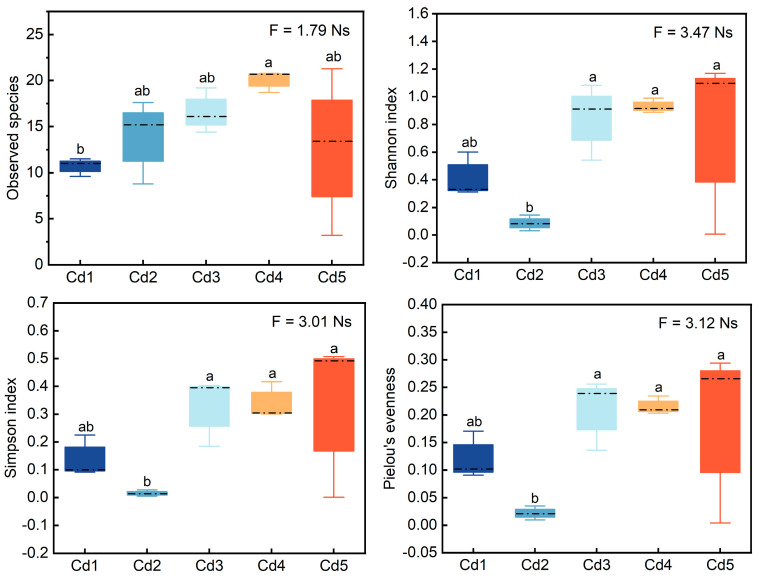
Good coverage representing integrity of sample coverage and α-diversity indices of culturable rhizobia from black locust under Cd exposure. Different lowercase letters indicate significant divergence (*p* < 0.05) between different treatments. Cd1–Cd5 represent 2, 4, 7, 15, and 20 mg Cd kg^−1^ dry weight soil, respectively. The * represents significance at *p* < 0.05, and Ns represents not significant.

The Goods coverage of *nifH* genes in culturable rhizobia at different Cd levels was over 99%, indicating that the sequencing structure could accurately reflect diversity of *nifH* genes in the cultures. Chao 1 and Observed_species indices showed that Cd3 and Cd4 treatments had higher community richness than other treatments, while the lowest richness was observed under Cd1 treatment (Figure 4). Shannon, Simpson, and Pielou’s evenness indices showed that Cd2 treatment had the lowest community diversity of *nifH* genes; additionally, three indices were higher under Cd3–Cd5 treatments than under Cd2 treatment (Figure 4). According to Pielou’s evenness index, the homogeneous of *nifH* genes in culturable rhizobia community under Cd2 treatments was lower than other treatments (Figure 4). Overall, the effect of Cd on alpha diversity was insignificant.

### 3.4. Community Structure of nifH Genes in Culturable Rhizobia

**Figure 5:**
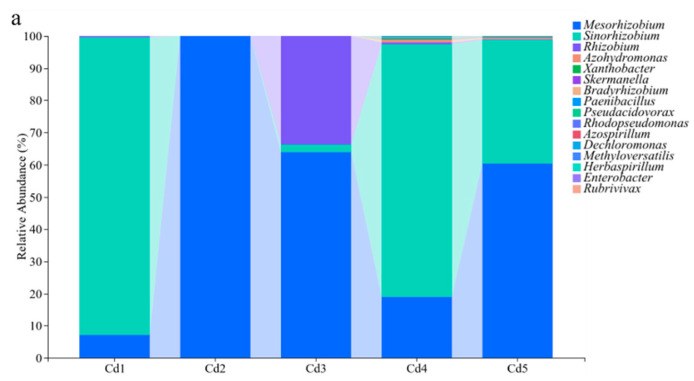

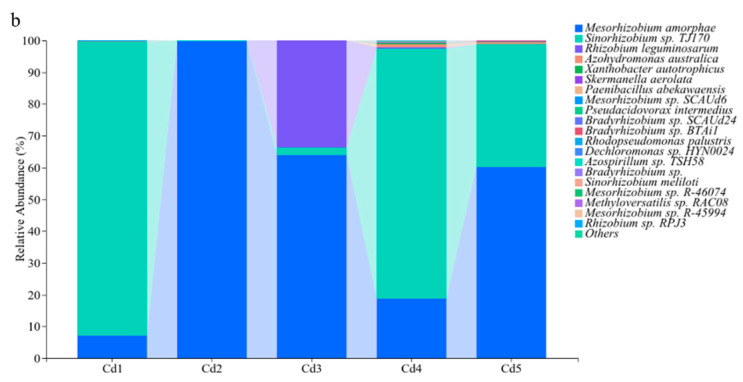
Relative abundance of culturable rhizobacteria at genus (**a**) and species (**b**) level from black locust grown in Cd-contaminated soils. Cd1–Cd5 represent 2, 4, 7, 15, and 20 mg Cd kg^−1^ dry weight soil, respectively.

At the genus level, only the top 10 genera in terms of relative abundance (>0.02%) were analyzed in this study. *Mesorhizobium* and *Sinorhizobium* were the first and second dominant genera in all treatments with relative abundance ranging from 7.18 to 99.92% and 0.08% to 92.52%, respectively (Figure 5a). Rhizobium was the third dominant genus with relative abundance ranging from 0.04% to 33.7%, and the relative abundance firstly increased and then decreased with increasing Cd (Figure 5a). The relative abundance of Rhizobium reached the highest at Cd3 level (Figure 5a), which suggested that the soil environment caused by Cd3 treatment might be more favorable for the survival of this genus. In addition, the relative abundance of seven genera including *Azohydromonas, Xanthobacter, Skermanella, Bradyrhizobium, Paenibacillus, Pseudacidovorax,* and *Rhodopseudomonas* increased under treatments Cd4 and Cd5 compared to other treatments (Figure 5a), suggesting that a higher Cd level might stimulate the survival of seven genera in soils.

In terms of relative abundance (>0.02%), only the top 10 species were detected, and the top two dominant species were *M. amorphae* and *S*. sp. TJ170 (Figure 5b). The highest abundance of the top two species appeared in different treatments: *M*. *amorphae* had the highest abundance under Cd2 treatment, and *Sinorhizobium* sp. TJ170 abundance was the highest at Cd1 level (Figure 5b). *R*. *leguminosarum* was the third dominant species with the highest relative abundance under Cd3 treatment (Figure 5b). In addition, *A*. *australica*, *X*. *autotrophicus*, *S*. *aerolata*, *P*. *abekawaensis*, *Mesorhizobium* sp. SCAUd6, *P*. *intermedius*, and *B*. sp. SCAUd24 abundance obviously increased under Cd4 and Cd5 treatments relative to other treatments (Figure 5b).

### 3.5. Characteristics of Culturable Rhizobia Communities in Terms of nifH Gene

#### 3.5.1. Principal Coordinate Analysis

**Figure 6:**
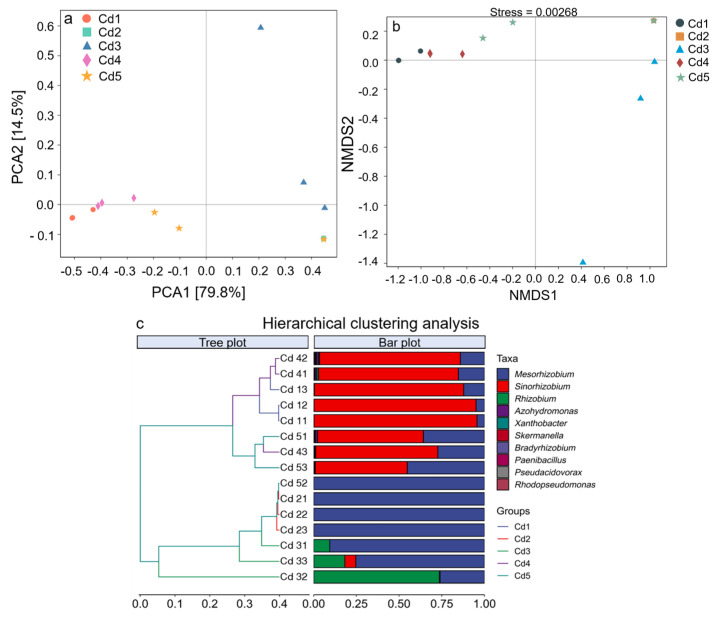
β-diversity of culturable rhizobacteria of black locust grown in Cd-contaminated soils. Panels (**a**–**c**) represent principal component analysis (PCoA), non-metric multidimensional scaling (NMDS), and cluster analysis (**c**) according to Bray–Curtis distance. Cd1, Cd2, Cd3, Cd4, and Cd5 treatments represent 2, 4, 7, 15, and 20 mg Cd kg^−1^ dry weight soil, respectively.

PCA1 and PCA2 explained the difference in treatments by 79.8% and 14.5%, respectively (Figure 6a), which could better distinguish the culturable rhizobia communities that could fix N under different Cd treatments. The community structure under treatments of Cd1, Cd4, and Cd5 was very similar according to the degree of aggregation. The PCA1 separated Cd2 treatment from treatments Cd1, Cd4, and Cd5, indicating that the community under Cd2 treatment was different from that under the other three treatments. Treatment Cd3 was farther away from other treatments, suggesting that the *nifH* gene community in culturable rhizobia under Cd3 treatment differed significantly from that under other treatments. Overall, there was greater divergence in the *nifH* gene community in culturable rhizobia under all treatments.

#### 3.5.2. Nonmetric Multidimensional Scaling

The spatial distribution of the culturable rhizobia that could fix N was well-reflected according to the stress value (0.00268) (Figure 6b). Treatments of Cd1, Cd4, and Cd5 had high aggregation, indicating that the difference in *nifH* genes in the culturable rhizobia community between these three treatments were little. However the community under Cd2 treatment was similar to that under Cd5 treatment, and Cd3 treatment was far away from other treatments, indicating an obvious heterogeneity of the *nifH* gene community between Cd3 and other treatments (Figure 6b).

#### 3.5.3. Community Clustering Analysis

The treatments could be mainly separated into two groups according to the UPGMA data. Treatments of Cd1, Cd4, and Cd5 were clustered into one unit, and Cd2 and Cd3 were clustered into another unit (Figure 6c). The two groups differed significantly in community structure and composition.

### 3.6. Genera Variation and Marker Genera Analysis

**Figure 7:**
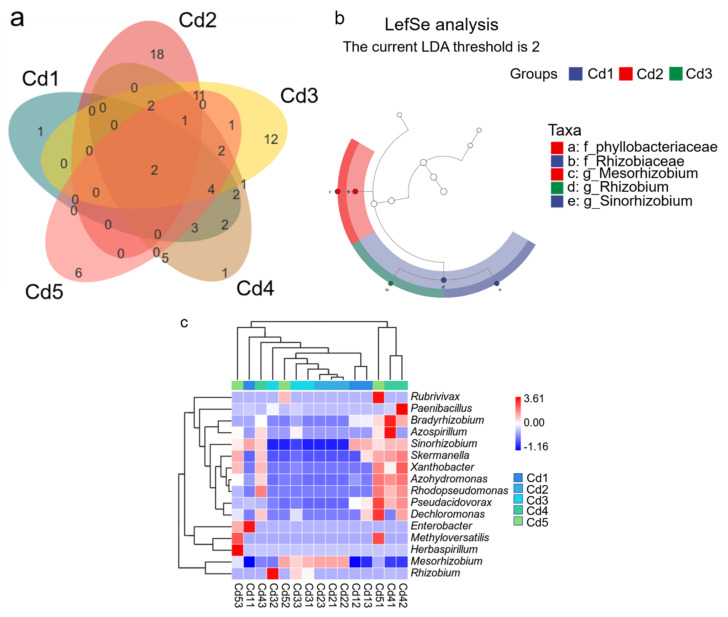
Venn diagram (**a**) according to the operational taxonomic units, LefSe analysis (**b**), and heatmap of the top 10 genera (**c**) of culturable rhizobia from black locust under Cd exposure. Cd1–Cd5 represent 2, 4, 7, 15, and 20 mg Cd kg^−1^ dry weight soil, respectively. Different circles from inside to outside represent phylum, class, order, family, and genus, and each node represents the species classification, and the node size represents the abundance level (LDA > 2) in panel (**b**).

A total of 74 OTUs were detected under five treatments according to Venn diagrams (Figure 6a). Five treatments shared two OTUs; the number of total OTUs under Cd1, Cd2, Cd3, Cd4, and Cd5 was 14, 34, 38, 25, and 24, respectively, while the specific OTUs were 1, 18, 12, 1, and 6, respectively (Figure 7a).

According to the heatmap, a total of 16 genera were clustered into three groups (Figure 7b); however, the treatments could be mainly clustered into two groups, Cd1, Cd2, Cd3, and Cd5 were mainly clustered into one group, and Cd4 was clustered separately into one group. The relative abundance of *Sinorhizobium* was higher under treatments of Cd1, Cd4, and Cd5 and the highest under Cd1 treatment; *Mesorhizobium* abundance was the highest under Cd2 treatment; and *Mesorhizobium* and *Rhizobium* abundance were the highest under Cd3 treatment (Figure 7b). Additionally, the relative abundance of *Mesorhizobium, Sinorhizobium*, and *Azohydromonas* was higher under treatments of Cd4 and Cd5.

The Cd1, Cd2, and Cd3 had genera with significant differences in relative abundance according to Lefse data (Figure 7c), and five divergence markers were identified. Two families were marked under treatments of Cd1 (*Rhizobiaceae*) and Cd2 (*Phyllobacteriaceae*). Additionally, *Sinorhizobium, Mesorhizobium*, and *Rhizobium* presented under Cd1, Cd2, and Cd3, respectively.

### 3.7. Relationship Between Diversity of nifH Gene in Culturable Rhizobia and Soil Factors

The soil pH affected the *nifH* gene diversity of culturable rhizobia according to a positive significant relationship between Shannon and Pielou’s evenness indices and pH (Table 1). Additionally, the total Cd in soils positively significantly influenced the diversity and evenness of the *nifH* gene in the culturable rhizobia according to the significant correlation between Shannon and Simpson indices and total Cd (Table 1).

*Mesorhizobium* abundance was negatively correlated (*p* < 0.05) with soil pH (Table 2). *Xanthobacter*, *Azohydromonas,* and *Rhodopseudomonas* were positively significantly correlated with total Cd, DTPA-Cd, and TC in soils (Table 2), and *Skermanella* abundance was positively significantly associated with total Cd and DTPA-Cd in soils (Table 2). Additionally, *Rhizobium* abundance was negatively correlated (*p* < 0.05) with soil C/N ratio, *Mesorhizobium* was negatively correlated (*p* < 0.05) with soil C/H ratio, and *Sinorhizobium* was positively correlated (*p* < 0.05) with soil C/H ratio (Table 2).

**Figure 8:**
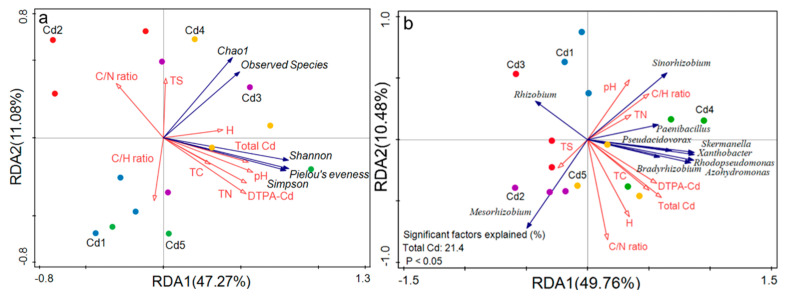
Redundancy analysis (RDA) between diversity and dominant genera of culturable rhizobia from black locust grown in Cd-contaminated soils. Panels (**a**,**b**) represent the diversity and dominant genera, respectively. TC, TN, TS, and H represented total carbon, total nitrogen, total sulfur, and H content, respectively. Cd1–Cd5 represent 2, 4, 7, 15, and 20 mg Cd kg^−1^ dry weight soil, respectively.

Soil factors explained 58.35% and 60.24% of the variations in the diversity and dominant genera of culturable rhizobia fixing N, respectively, based on the RDA data (Figure 8a,b). The total Cd positively significantly affected *Mesorhizobium*, *Rhizobium*, *Bradyrhizobium*, *Azohydromonas*, *Rhodopseudomonas*, *Xanthobacter*, *Skermanella*, *Pseudacidovorax*, and *Paenibacillus* and negatively significantly influenced *Sinorhizobium* (Figure 8b).

## 4. Discussion

In this study, the significant difference in *nifH* diversity and evenness of culturable rhizobia between Cd2 and Cd4 were associated with soil pH and total Cd according to Pearson analysis. The changes in soil pH and total Cd might be caused by black locust growth. Our previous studies indicated that black locust roots released lots of compounds, such as phenolic acids, soluble sugars, amino acids, and flavonoids, into soils during growth [31,32,33,34], which could change soil pH and total Cd. It is well known that pH and heavy metals are significant environmental factors in microbial survival. Thus, the difference in total Cd and pH between Cd2 and Cd4 caused by seedling growth led to a significant divergence of *nifH* gene diversity and evenness. Additionally, Cui [35] found that the diversity of inter-root bacteria obviously increased with soil Cd level, which also supported our results. Although Cd uptake by plant roots might affect the formation of root nodules by inhibiting rhizobia reproduction and the synthesis or activity of *RinRK1* and Flotillin 1 playing a crucial role in promoting symbiotic infection [36], the insignificant change in *nifH* diversity suggested that Cd in the roots might have little effect on the colonization of culturable rhizobia in the roots in this study. Additionally, previous studies indicated that Cd uptake by black locust was much lower than Cd remaining in soils and mainly existed in hyphae and the vesicular structure of arbuscular mycorrhizal fungi in the roots [37,38,39,40], which suggested that Cd in the roots might have little effect on rhizobia colonization in this study. Of course, it remains to thoroughly explore the effect of Cd in hyphae and vesicular structure on symbiotic nitrogen fixation colonization of nodules in the future. Increased C and N in soils under Cd3–Cd5 treatments relative to Cd2 might be caused by more exudates released by the roots due to high Cd levels. Additionally, Cd exerts inhibitory impacts on plant physiological metabolism and root development, thereby impairing nutrient absorption and utilization capacity [41], resulting in the accumulation of residual nutrients within the soil matrix, which might be a reason for higher C and N content in soils.

Many studies indicated that *Mesorhizobium* was the absolute dominant genus in rhizobia from black locust [42,43]; however, *Mesorhizobium* only appeared under the treatments of Cd2, Cd3, and Cd5 with the first absolute dominant species, and the relative abundance of the top three absolute dominant genera (*Mesorhizobium*, *Sinorhizobium* and *Rhizobium*) was different between five treatments, which suggested that the symbiosis of black locust roots and the presence of Cd might be the main reason for the variety of dominance in the absolute dominant genus. Cadmium might affect survivable rhizobia species in soils according to the RDA data, which led to the difference in rhizobia species in the roots between five treatments. Generally, the soil Cd level showed an obvious effect on culturable rhizobia community structure. The presence of *Mesorhizobium*, *Sinorhizobium,* and *Rhizobium* under five treatments indicated that the three genera could live under Cd exposure, which suggested that the three genera might be used for remediation of Cd-contaminated soils in the future. *Mesorhizobium*, *Sinorhizobium*, and *Rhizobium* have been found to have the ability to resist Cd and other heavy metals due to producing extracellular polymers, etc. [44,45,46,47], which may be the reason for the presentence of these genera under all treatments. As an important bacterium involved in nitrogen fixation, *Mesorhizobium* played a crucial role in ameliorating heavy metal (Cd, copper and zinc)-polluted soils [47,48], which agreed with our results that *Mesorhizobium* could tolerate Cd in soils. However, *Sinorhizobium* was the absolute dominant genus under high Cd (Cd4 and Cd5) levels, indicating that the tolerance of *Sinorhizobium* (such as *S*. sp. TJ170) was superior to *Mesorhizobium*. Additionally, the highest *Sinorhizobium* abundance under Cd1 (2 mg Cd kg^−1^ dry soil) indicated that it might be less sensitive to Cd stress relative to other genera. The above suggested that *Sinorhizobium* might be used for the remediation of plant microbes in lower and higher Cd-contaminated soils. However, the sensitiveness of *Mesorhizobium* to Cd was higher according to the lowest abundance under Cd1 treatment, which suggested that *Mesorhizobium* might be used as an indicator of lower Cd-contaminated soils. A higher abundance of *Mesorhizobium* (such as *M. amorphae*) relative to *Sinorhizobium* under treatments of Cd2 and Cd3 indicated the sensitiveness of *Mesorhizobium* was lower, which suggested that *Mesorhizobium* might be applied for the remediation of plant microbe in medium Cd (4~7 mg Cd kg^−1^ dry soil)-contaminated soils. A previous study has indicated that microorganisms make them resistant to heavy metals through a variety of mechanisms [49]; thus, *Mesorhizobium*, *Sinorhizobium*, and *Rhizobium* also might rely on efflux pumps, chelating molecules (GSH), and antioxidant defenses to tolerate Cd in this study. In addition, *Mesorhizobium* relies more on EPS and phytochelatorin synergistic [50], *Sinorhizobium* emphasizes the high efficiency of membrane structure adjustment and efflux systems [51], and *Rhizobium* often uses plasmid-encoding genes and cell wall adsorption and has a significant synergistic mechanism with the host [52], which might be the reasons for the resistance of three genera to Cd stress. In addition, *Skermanella* was identified to be sensitive to Cd [53], which might explain the obvious difference in relative abundance of this genus under different Cd treatments in this study. Overall, Cd caused some changes in α- and β-diversity of the rhizobia community according to the significant correlation between Shannon and Pielou’s evenness indices and total Cd in soils and the order of dominant genera in community between different treatments.

Higher *r* (*S*. sp. TJ170) abundance under Cd1, Cd4, and Cd5 treatments might be caused by higher soil C/H ratio according to their positive significant correlation. Higher soil C/H ratio benefits plant growth, which might provide organic nutrients for *Sinorhizobium* (*S*. sp. TJ170). The similar community structure under treatments of Cd1, Cd4, and Cd5 according to PCoA, NMDS, and hierarchical cluster data indicated that 2, 15, and 20 mg Cd kg^−1^ dry soil showed similar effects on culturable rhizobia, which suggested that soil Cd level almost never caused the disappearance of culturable species. As the top one dominant genus (species) under treatments of Cd2 and Cd3, *Mesorhizobium* (*M. amorphae*) might be sensitive to soil Cd concentration. Additionally, *Azorhizobium*, *Xanthobacter*, and *Skermanella* were positively significantly correlated with DTPA-Cd and TC in addition to being significantly affected by total Cd. Generally, Cd showed an obvious negative effect on culturable rhizobia community structure.

Relative to non-cultured rhizobia from cultivated and wild halophytic legumes [11], *Enterobacter* and *Stenotrophomonas* were not found in our cultures in addition to the absolutely dominant genus (*Mesorhizobium*, *Sinorhizobium*, and *Rhizobium*), which suggested that not all rhizobia could be cultured. However, the culturable rhizobia in this study provided good microbial resources for the remediation of plant microbes on Cd-contaminated soils according to host-mixed symbiotic relationship (i.e., one rhizobia strain can be tumoring with a variety of legumes for nitrogen fixation) [54], which should be selected for Cd phytoremediation by analyzing nitrogen fixation capacity and the ability to produce indoleacetic acid, gibberellin, siderophores, and lyases, which promote plant growth in the future.

## 5. Conclusions

Overall, cadmium in soils had an obvious negative effect on the community structure of culturable rhizobia from black locust seedlings, which might be associated with inhibition of Cd on microbial growth. *Mesorhizobium*, *Sinorhizobium,* and *Rhizobium* were the absolute dominant genera in culturable rhizobia communities across all Cd levels. Furthermore, *Mesorhizobium* and *Sinorhizobium* were more sensitive to Cd than other culturable rhizobia. Additionally, the total Cd in soils distinctly affected the relative abundance of *Azohydromonas, Xanthobacter, Skermanella, Bradyrhizobium, Paenibacillus, Pseudacidovorax*, and *Rhodopseudomonas*. The soil pH negatively significantly affected *Mesorhizobium* abundance in culturable rhizobia. The results will provide us a new insight into the selection of excellent strains that can promote phytoremediation of heavy-metal-contaminated soils.

## Figures and Tables

**Table 1:** Pearson correlation between diversity index of culturable rhizobia community from black locust and soil physicochemical factors.

Index	pH	Total Cd	DTPA-Cd	C/N Ratio	C/H Ratio	TS	H	TN	TC
Chao 1	0.123	0.174	0.208	−0.034	−0.434	0.278	0.179	0.003	−0.076
Observed species	0.204	0.289	0.188	0.095	0.017	0.086	0.232	0.157	0.212
Shannon	0.528 *	0.539 *	0.453	−0.292	0.042	−0.019	0.319	0.506	0.300
Simpson	0.436	0.580 *	0.497	−0.272	−0.025	−0.090	0.334	0.475	0.277
Pielou’s evenness	0.54 *	0.488	0.422	−0.372	0.031	−0.055	0.239	0.489	0.223

Note: The * indicates significance at *p* < 0.05.

**Table 2:** Pearson correlation between culturable dominant rhizobia from black locust and soil physicochemical properties.

Genus	pH	Cd	DTPA-Cd	C/N Ratio	C/H Ratio	TS	H	TN	TC
*Mesorhizobium*	−0.529 *	0.022	0.026	0.493	−0.533 *	0.299	0.256	−0.344	−0.054
*Sinorhizobium*	0.454	0.033	0.006	−0.220	0.634 *	−0.246	−0.247	0.191	0.115
*Rhizobium*	0.089	−0.149	−0.093	−0.546 *	−0.299	−0.072	−0.011	0.275	−0.167
*Azohydromonas*	0.223	0.648 **	0.553 *	0.286	0.361	−0.182	0.494	0.354	0.626 *
*Xanthobacter*	0.176	0.703 **	0.629 *	0.170	0.252	−0.288	0.434	0.331	0.515 *
*Skermanella*	0.266	0.645 **	0.562 *	0.178	0.262	−0.233	0.330	0.216	0.427
*Bradyrhizobium*	0.324	0.372	0.257	0.221	0.286	0.026	0.343	0.235	0.451
*Paenibacillus*	0.271	0.215	0.120	−0.146	0.226	−0.087	0.070	0.216	0.184
*Pseudacidovorax*	0.175	0.446	0.418	0.186	0.426	−0.101	0.308	0.309	0.500
*Rhodopseudomonas*	0.160	0.635 *	0.523 *	0.284	0.397	−0.241	0.501	0.380	0.653 **

Note: ** and * indicate significance at *p* < 0.01 and *p* < 0.05, respectively.

## Data Availability

All data analyzed are included within the article.
